# Production and clinical evaluation of breast lesion skin markers for automated three-dimensional ultrasonography of the breast: a pilot study

**DOI:** 10.1007/s00330-020-06695-y

**Published:** 2020-02-14

**Authors:** Leon de Jong, Marcel K. Welleweerd, Jan C.M. van Zelst, Francoise J. Siepel, Stefano Stramigioli, Ritse M. Mann, Chris L. de Korte, Jurgen J. Fütterer

**Affiliations:** 1 Department of Radiology and Nuclear Medicine, Radboud University Medical Center, P.O. Box 9101, internal postal code 766, 6500 HB Nijmegen, The Netherlands; 2 Department of Robotics and Mechatronics, University of Twente, Enschede, The Netherlands

**Keywords:** Diagnostic imaging, Fiducial markers, Breast ultrasonography, Three-dimensional image, Breast cancer

## Abstract

**Objectives:**

Automated ultrasound of the breast has the advantage to have the whole breast scanned by technicians. Consequently, feedback to the radiologist about concurrent focal abnormalities (e.g., palpable lesions) is lost. To enable marking of patient- or physician-reported focal abnormalities, we aimed to develop skin markers that can be used without disturbing the interpretability of the image.

**Methods:**

Disk-shaped markers were casted out of silicone. In this IRB-approved prospective study, 16 patients were included with a mean age of 57 (39–85). In all patients, the same volume was imaged twice using an automated breast ultrasound system, once with and once without a marker in place. Nine radiologists from two medical centers filled scoring forms regarding image quality, image interpretation, and confidence in providing a diagnosis based on the images.

**Results:**

Marker adhesion was sufficient for automated scanning. Observer scores showed a significant shift in scores from excellent to good regarding diagnostic yield/image quality (*χ*^2^, 15.99, *p* < 0.01), and image noise (*χ*^2^, 21.20, *p* < 0.01) due to marker presence. In 93% of cases, the median score of observers “agree” with the statement that marker-induced noise did not influence image interpretability. Marker presence did not interfere with confidence in diagnosis (*χ*^2^, 6.00, *p* = 0.20).

**Conclusion:**

Inexpensive, easy producible skin markers can be used for accurate lesion marking in automated ultrasound examinations of the breast while image interpretability is preserved. Any marker-induced noise and decreased image quality did not affect confidence in providing a diagnosis.

**Key Points:**

*• The use of a skin marker enables the reporting radiologist to identify a location which a patient is concerned about.*

*• The developed skin marker can be used for accurate breast lesion marking in ultrasound examinations.*

## Introduction

Since automated breast ultrasound (ABUS) scanners have been introduced to the market, the value and potential of these scanners is continuously being explored in clinical practice. Current ABUS offers high-quality volumetric image data that can be assessed in the 3D orthogonal imaging planes that are familiar to the radiologist. Examples of applications of ABUS in both clinical practice and clinical research are adjunct modality to mammography, lesion classification, therapy response, and second-look examinations [1, 2]. Handheld ultrasound (HHUS) as an adjunct to mammography showed improved detection of small early-stage invasive breast cancers when compared with mammography alone [3–8]. However, offering handheld ultrasound screening requires substantial resources and has a known operator dependency [9]. ABUS overcomes some of the limitations of HHUS, e.g., costs and operator dependency [10]. With minimal training, non-sonographers can perform ABUS examinations after which a dedicated breast radiologist can perform batch or remote reporting. As a consequence, the interaction between patient and radiologist is lost. Feedback about focal abnormalities (e.g., palpable lesions) is therefore also not available. While for other imaging modalities effective skin markers are available and well known, for ultrasound, such a marker is lacking. Radiopaque markers for X-ray-based mammography can be used to annotate a variety of abnormalities and are even included in widely accepted guidelines for mammography [11–13]. In MRI, oil-based vitamin E capsules can be used for a wide range of purposes that require location marking [14]. Analogue to these markers for other modalities, we aim to preserve and provide relevant information on focal abnormalities to the radiologist by introducing a skin marker for ultrasound examination. The site to which the skin marker refers is based on input from the patient or based upon clinical examination by a physician or the technician. From a patient perspective, this enables patients to be more involved in ABUS screening by indicating the location that raises concerns. In this study, we propose a method to produce ultrasound-compatible skin markers and assess effects on image quality and interpretation using expert observers from two medical centers.

## Methods

### Patient inclusion

To obtain a representative and heterogeneous population, patients planned for mammography examination for clinical indications were asked to participate in this study. Patients with any prior breast surgery or intervention were not eligible. Ethical approval was given by the regional medical ethics review committee and written informed consent was received from all participants. In total, 16 patients were included.

### Marker production

Markers were casted out of silicone. A mold was 3D printed using a Fortus 250MC (Stratasys, Ltd.) printer with a rubber-like print material, Tango (Stratasys, Ltd.). This resulted in a flexible mold that allowed releasing the markers from the mold after curing. The resulting marker is a disk of 2 mm thick and 5 mm in diameter. Initially, a marker of 10 mm in diameter was produced and was only used on the first two patients. The marker’s material is Ecoflex™ GEL (Smooth-on Inc.) which is commercially available and tested skin safe that conform with the OECD TG 439 test guidelines which are closely related to the ISO 10993 standards for medical devices [15]. For this pilot study, we rely on these test results and patients were asked to report any adverse events. The two components of uncured silicone were mixed, stirred, and carefully poured into the mold. Any excess material was scraped off. The markers were left to cure for 4 h of which the first hour in a vacuum chamber at 100 mbar to extrude any trapped air. After curing, the markers were removed from the mold and stored on a plastic sheet for later application. The permanently sticky silicone allowed for long-term storage of the marker without changes to geometrical or acoustic properties.

### Image acquisition

Ultrasound examinations were performed with the patient in supine position using an Acuson S2000 ABVS system (Siemens Healthineers). Patients with a (suspected) focal abnormality were asked to point out the exact location where the technician should place the marker. If the patient did not have a focal abnormality, the marker was placed at an arbitrary location. The same breast volume was imaged twice: once with and once without a marker in place. The ABUS interface offers cup size-based presets that alter the ultrasound settings: frequency, depth, and focal zones. The best corresponding preset was chosen by the technician according to routine acquisition protocols. User feedback was collected from technicians to assess the feasibility of implementing the marker in the current clinical workflow. Questions were formulated regarding marker handling, functionality, and patient discomfort and were scored on a 5-point Likert scale: 1, strongly disagree; 2, disagree; 3, neutral; 4, agree; 5, strongly agree. See Table 1 for the full statements.

**Table 1:** Technician (*n* = 3) user feedback with Likert scoring scale: strongly disagree (SD), disagree (D), neutral (N), Agree (A), and strongly agree (SA)

Technician feedback					
Statement	SD	D	N	A	SA
The marker is easy to manually grab and hold		■■		■	
Marker adhesion is sufficient to apply onto the skin			■■	■	
The marker can be placed accurately on the desired location		■		■	■
The marker stays in place during scanning				■■	■
The marker can be easily removed from the skin				■	■■
The required extra time needed for marker placement is negligible w.r.t. scanning without marker				■■■	
It is feasible to implement the marker in the current clinical workflow		■		■	■
The marker induces no added discomfort to the patient w.r.t. scanning without a marker					■■■

### Observer assessment

The influence of the marker on image quality, interpretability, and marker detectability was qualitatively analyzed in 16 patients by nine radiologists from an academic (*n* = 4) and a regional (*n* = 5) breast care clinic, resulting in 144 observations with marker and 144 observations without a marker. Radiologist experience levels were fellow (*n* = 2), 5 ≤ 10 years (*n* = 2), and > 10 years (*n* = 5). In contrast to the radiologists from the academic center, the radiologists from the regional care clinic were unfamiliar with assessment of volumetric breast ultrasound images. Images were assessed using a dedicated image viewing platform: sUSBA VA25A (Siemens Healthineers). An example image was provided to the observers to indicate the typical aspect of a marker in the coronal plane. Assessment was performed in two rounds with a minimal interval period of 2 weeks. The scans with and without a marker were randomized. In each round, roughly half of the cases were read without a marker in place, whereas the other half were read with the marker in place. Cases that were presented with a marker in the first round were shown without a marker in the second round and vice versa. In neither round, prior knowledge was provided whether or not a scan contained a marker.

### Statistical analysis

Pearson chi-square test was used to test associations between marker presence and observer scores using SPSS software, version 25(IBM®).

### Accuracy measurement

**Fig. 1:**
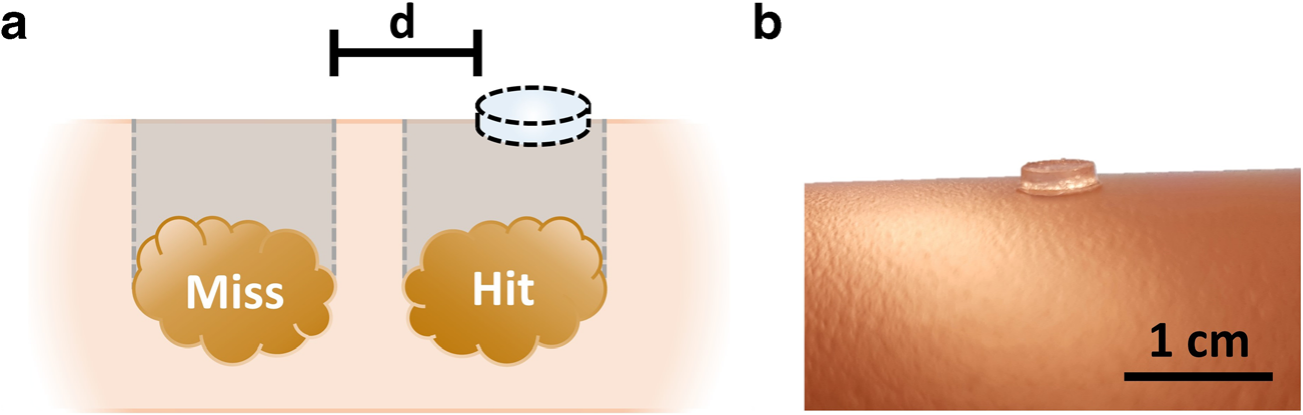
Schematic representation of hit and miss classification (**a**). Classification depends on the surface projection of a lesion to the skin surface. If the lesion surface projection hits the marker surface, this is classified as a hit. In case of a miss, the shortest distance of the projection to the edge of the marker is defined as the distance error “d”. A photograph showing the appearance of the marker (**b**)

Placement accuracy was assessed in patients with focal abnormalities that were confirmed on ultrasound imaging. Marker placement was deemed accurate when the marker was placed in vertical alignment with the lesion. In case of misplacement, the accuracy error is defined as the minimal distance from the marker to the skin surface projection of the lesion in the coronal plane (see Fig. 1).

## Results

### Feasibility clinical workflow

Overall, user feedback supports that the marker satisfied all user aspects except for statements concerning manual manipulation (Table 1). This could be ascribed to the small size and sticky surface of the marker. This caused the marker to stick not only to the patient but also to the fingers of the technician. In all cases, the marker stayed in place during scanning. Two out of three users (strongly) agreed that it is feasible to implement the marker in the current clinical workflow; the third user disagreed with this statement.

### Observer assessment

**Fig. 2:**
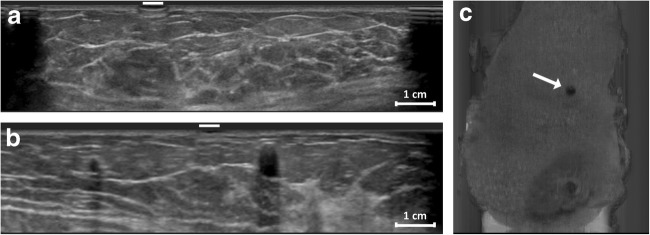
Orthogonal imaging planes at marker position: transversal acquisition plane (**a**), sagittal reconstruction (**b**), and coronal reconstruction (**c**). The marker is indicated by a white line or arrow. The aspect of the marker is most prominent in the superficial coronal reconstruction

In 94% of scans containing a marker, the marker was successfully detected (Fig. 2). In 5% (13 out of 288) of all cases, observers scored “uncertain of marker presence” in which case 7 of these cases actually contained a marker and 6 cases did not. From all scans containing a marker (*n* = 144), 6% was not detected. The group without a marker (*n* = 144) contained 7% false positives. Based on the qualitative reporting of the radiologists, statements “1. Image quality is according to my expectations” (*χ*^2^, 4.79, *p* = 0.31) and “2. I feel confident providing a diagnosis based on these images” (*χ*^2^, 5.99, *p* = 0.20) were not influenced by the presence of the marker.

**Fig. 3:**
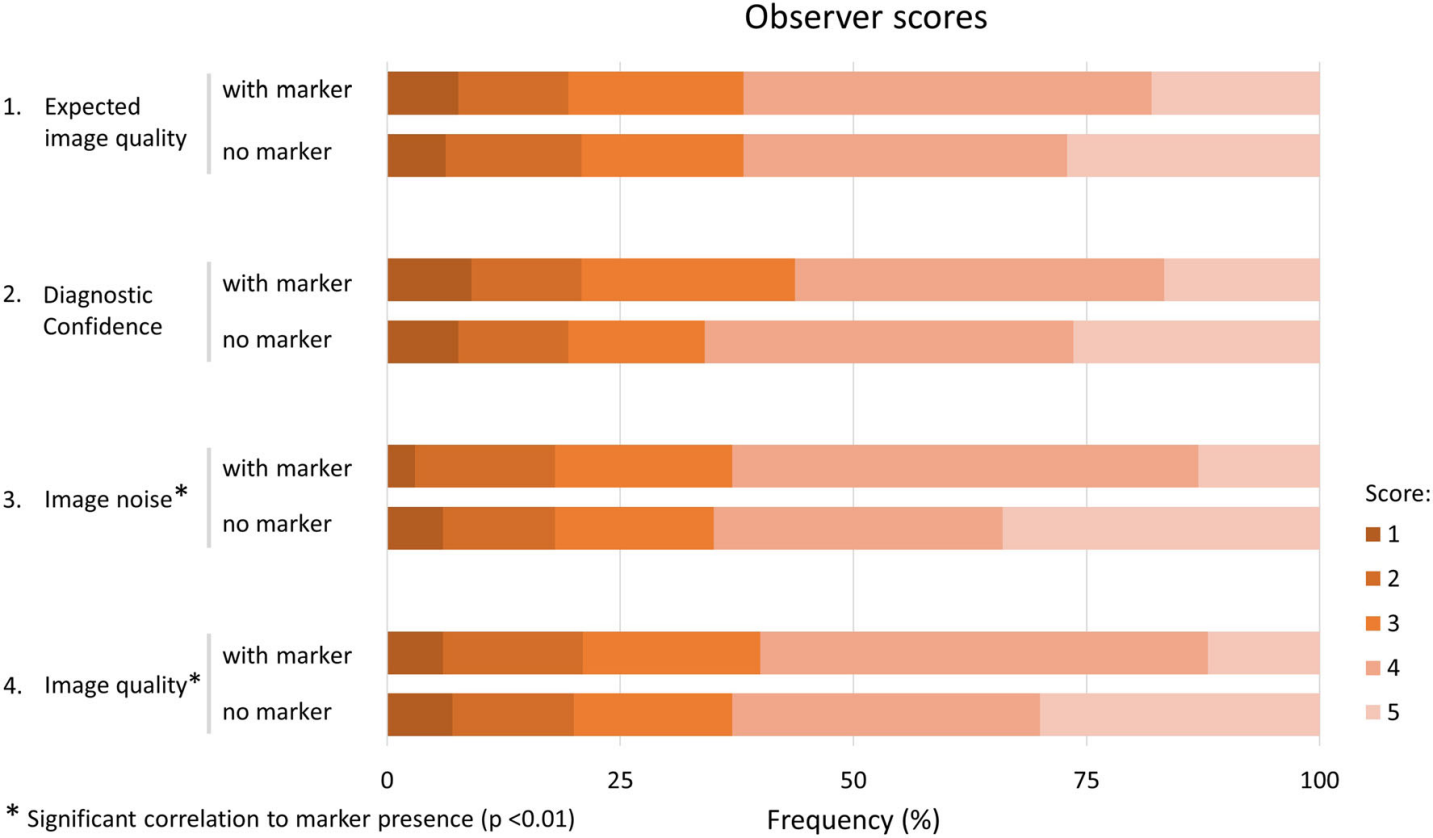
Frequency distribution of observer scores. Definition of scores for statement “1. Image quality is according to my expectations” and “2. I feel confident providing a diagnosis based on these images”: 1, strongly disagree; 2, disagree; 3, neutral; 4, agree; 5, strongly agree. Definition of scores for statement “3. Image noise“: 1, major, no diagnosis possible; 2, noise impeding diagnosis; 3, moderate, sufficient for diagnosis; 4, minor, no influence on diagnosis; 5, none perceivable. Definition of scores for statement “4. Diagnostic yield/Image quality“: 1, poor, no diagnosis possible; 2, low, confidence in making diagnosis degraded; 3, moderate but sufficient for diagnosis; 4, good; 5, excellent

**Fig. 4:**
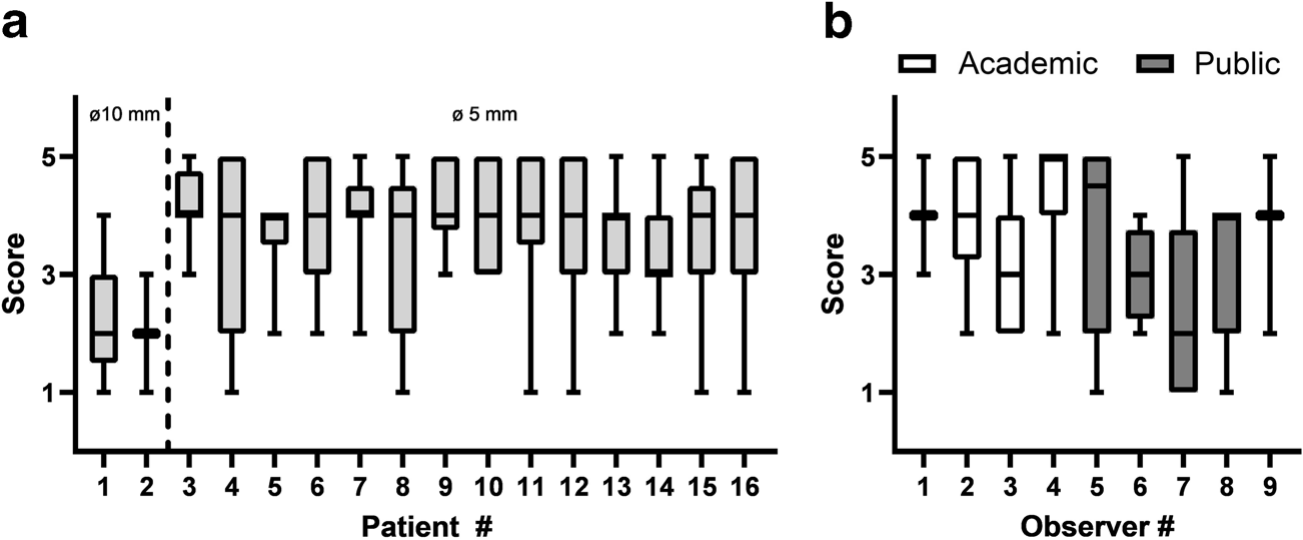
Frequency distributions on statement “image noise induced by the marker does not influence image interpretation.” Scores meaning 1, strongly disagree; 2, disagree; 3, neutral; 4, agree; 5, strongly agree. In this distribution, only scores of scans with a marker were included. Only the first two patients were scanned with the ø 10-mm marker

Statements 3 and 4, concerning “3. image noise” (*χ*^2^, 21.20, *p* < 0.01) and “4. Diagnostic yield/image quality” (*χ*^2^, 15.99, *p* < 0.01), were affected by marker presence. For both items, a shift in scores from 5 to 4 was observed, meaning a decrease in quality from excellent to good, and an increase in image noise from none perceivable to minor not influencing diagnosis, respectively (Fig. 3). Median scores on the final marker (ø 5 mm) showed agreement with the statement “Image noise induced by the marker does not influence image interpretation” for 13 out of 14 patients (see Fig. 4a). The size of the marker clearly had an impact on image interpretation as the bigger marker, used in the first two patients, induced major artifacts (Fig. 4a).

### Placement accuracy

**Fig. 5:**
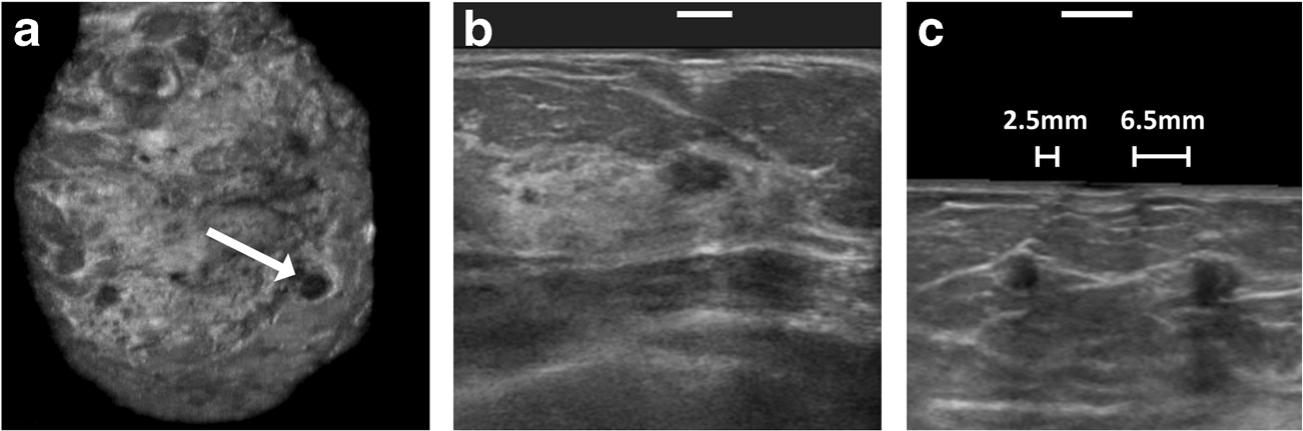
The coronal (**a**) and transversal image (**b**) of a patient with a marker and cyst that was classified as a hit. The white arrow depicts the cyst with a nominal diameter of 8 mm at 11.5 mm depth. The same cyst can be seen in the transversal image in which the marker position is indicated with a white line. The oblique plane of another patient shows a miss and the corresponding distance errors of in the two small cysts with nominal diameters of 2.5 mm and 6.5 mm at 7 mm depth (**c**)

Four patients had confirmed abnormalities; these were all cysts. Accuracy assessment showed that two markers were accurately placed and two markers actually missed the lesion. One miss was potentially caused by an abnormality that consisted of two neighboring small cystic lesions in which a palpable center was difficult to point out (Fig. 5c). The other miss had a distance error of 1.5 mm from a cyst with a nominal diameter of 11 mm at 7 mm depth.

### Safety

In this study, no adverse events were reported. All technicians strongly agreed with the statement that no additional patient discomfort was induced by the marker compared with conventional scanning.

## Discussion

The intended goal of a skin marker is to provide additional information to the radiologist, when there is no direct contact between the radiologist and the patient. Given the increasing workload for radiologists, patient contact is likely to decrease further and the use of a marker may increase efficiency. In this study, we show that it is feasible to use a small silicone marker for this purpose in ABUS acquisitions. Based on this proof of concept, we intend to evaluate the marker in a larger population in the near future. The aim of this study should include assessment of the effects on screening efficacy and involve patients and their experience regarding discomfort and confidence in screening. Second, this larger population allows for adequate validation of the presumed safety of the silicone material for this application.

An optimal marker would be clearly visible without affecting image quality and interpretability. Clear visibility was reached due to the hypoechogenic appearance of the marker. However, to maintain image quality and interpretability, the acoustic properties of the marker should be close to the properties of breast tissue. However, silicone differs 540 ms^−1^ in speed of sound and 0.6 MRayl in acoustic impedance with respect to breast tissue [16]. The mismatch in acoustic impedance causes less energy to be transmitted into the breast tissue. The difference in speed of sound influences ultrasonic beamforming possibly affecting the lateral resolution. The effects of this material to tissue mismatch may have contributed to the significant relations between marker presence and observer scores on image quality and noise. A material that better matches the acoustic properties of breast tissue may therefore further reduce the artifacts. Little is known about materials that can be used for the specific purpose of this study. Still, several well-known tissue-mimicking materials other than silicone are often used as a phantom material: gelatin, agar, polyvinyl alcohol (PVA) polymers [17–20]. Even though these materials better match the acoustic properties of tissue, they all lack one or more of the properties needed for a skin marker, e.g. storage of the marker without degrading or the possibility to safely stick the marker to skin. Therefore, we believe that the silicone used in this study offers a good trade-off regarding the desired marker properties: low-cost material, easy production, storage possibility, sufficient skin adhesion, safe for skin application, clear visibility, and minimal artifact induction. Considering hygiene and loss of stickiness after single use, the markers should be considered disposable. Costs are negligible as the printed mold can be reused and the silicone costs several tens of US dollars to make millions of markers.

User feedback from technicians showed that the ability of manually handling the marker needs improvement. One of the three technicians experienced difficulties during handling of the marker and therefore disagreed with the statement that it is feasible to implement the marker in the current clinical routine. A larger marker would be easier to handle. However, increasing the marker size negatively affects interpretability as was seen in the first two patients, and is therefore not an appropriate solution. An alternative solution would be to cast the marker in two layers, the sticky silicone being the bottom layer and a second layer of a non-sticky silicone that bonds to the sticky layer, e.g., Ecoflex™ 0–10 (Smooth-on Inc.). This way, the marker would only stick to the patient and not to the hand of the technician.

Since our study focused on the effect of the marker on image quality and interpretability, we did not select patients for the presence of abnormalities. Consequently, we included only four patients with a focal lesion that was confirmed on ultrasound. While we believe the observed placement accuracy of the markers is sufficient for clinical practice, further studies to provide exact metrics on placement precision are required.

Software packages that come with ABUS systems allow digital annotation and location marking (e.g., marking the nipple). Therefore, one could perceive that technologists could digitally annotate the location of an abnormality on the scan. This would preclude the need of a physical marker. However, this requires adequate interpretation by the technician of the location of a focal abnormality and the appearance of this location within the volumetric images, which is non-trivial. We would therefore rather propose to apply the physical marker over the lesion location, and have the technologist annotate the presence of the marker. This would also prevent potential overlook of the marker by a radiologist and would solve the few false positive interpretations observed in our study, where radiologists pointed out markers that were not physically there (likely caused by small air bubbles with a similar appearance in the coronal plane).

In conclusion, inexpensive, easy producible skin markers can be used for accurate lesion marking in ABUS examinations while image interpretability is preserved. Any marker-induced noise and decreased image quality did not affect confidence in providing a diagnosis.

## Abbreviations

3D: Three-dimensional
ABUS: Automated breast ultrasound
HHUS: Handheld ultrasound
MRI: Magnetic resonance imaging
